# PBDE flame retardants, thyroid disease, and menopausal status in U.S. women

**DOI:** 10.1186/s12940-016-0141-0

**Published:** 2016-05-24

**Authors:** Joseph G. Allen, Sara Gale, R. Thomas Zoeller, John D. Spengler, Linda Birnbaum, Eileen McNeely

**Affiliations:** Department of Environmental Health, Harvard T. H. Chan School of Public Health, 401 Park Drive, Boston, MA 02215 USA; University of Massachusetts Amherst, Amherst, MA USA; National Cancer Institute/NIEHS, Research Triangle Park, NC USA

**Keywords:** Endocrine disruptors, Polybrominated diphenyl ethers, PBDE, Thyroid, Menopause

## Abstract

**Background:**

Women have elevated rates of thyroid disease compared to men. Environmental toxicants have been implicated as contributors to this dimorphism, including polybrominated diphenyl ethers (PBDEs), flame retardant chemicals that disrupt thyroid hormone action. PBDEs have also been implicated in the disruption of estrogenic activity, and estrogen levels regulate thyroid hormones. Post-menopausal women may therefore be particularly vulnerable to PBDE induced thyroid effects, given low estrogen reserves. The objective of this study was to test for an association between serum PBDE concentrations and thyroid disease in women from the United States (U.S.), stratified by menopause status.

**Methods:**

Serum PBDE concentrations (BDEs 47, 99, 100 and 153) from the National Health and Examination Survey (NHANES) and reports on thyroid problems were available in the NHANES 2003–2004 cycle. Odds ratios (ORs) were calculated using multivariate logistic regression models accounting for population-weighted survey techniques and controlling for age, body mass index (BMI), education, smoking, alcohol consumption and thyroid medication. Menopause status was obtained by self-reported absence of menstruation in the previous 12 months and declared menopause.

**Results:**

Women in the highest quartile of serum concentrations for BDEs 47, 99, and 100 had increased odds of currently having thyroid disease (ORs: 1.5, 1.8, 1.5, respectively) compared to the reference group (1st and 2nd quartiles combined); stronger associations were observed when the analysis was restricted to postmenopausal women (ORs: 2.2, 3.6, 2.0, respectively).

**Conclusion:**

Exposure to BDEs 47, 99, and 100 is associated with thyroid disease in a national sample of U.S. women, with greater effects observed post-menopause, suggesting that the disruption of thyroid signaling by PBDEs may be enhanced by the altered estrogen levels during menopause.

**Electronic supplementary material:**

The online version of this article (doi:10.1186/s12940-016-0141-0) contains supplementary material, which is available to authorized users.

## Background

Fire safety standards promulgated in California in the late 1970’s have led to the proliferation of chemical flame retardants (FRs) used in consumer products worldwide [1]. Brominated flame retardant compounds are used as additives in products at up to 20 % by weight of the product [2]. Precisely because they are used as additives, rather than covalently bound to a polymer matrix, these halogenated FRs can and do migrate from their source products into the indoor and outdoor environment [3]. In fact, the global use of one class of environmentally-persistent flame retardant chemicals over a 35-year period, polybrominated diphenyl ethers (PBDEs), has led to their near ubiquitous presence in animals, abiotic matrices and food, as well as in air, dust and surfaces of indoor environments [4–17].

PBDEs share a similar structure to polychlorinated biphenyls (PCBs), polybrominated biphenyls (PBBs), tetrabromobisphenol A (TBBPA), triclosan, and the halogenated-phenyl ring of thyroid hormone thyroxine (T_4_), with the potential for 209 different congeners based on the number and position of the bromine within the aromatic rings. PBDEs were manufactured in three commercial products—PentaBDE, OctaBDE, DecaBDE—each of which is dominated by one or more congeners (BDEs 47 (2,2′4,4′-tetra-bromodiphenyl ether), 99 (2,2′4,4′5-penta-bromodiphenyl ether), 100 (2,2′,4,4′,6-penta-bromodiphenyl ether); BDE 183 (2,2′,3,4,4′,5′,6-hepta-bromodiphenly ether); BDE 209 (deca-bromodiphenyl ether), respectively). The use of PBDEs as flame retardants have been phased out or banned in some countries, and updated California fire safety standards no longer require PBDEs or other flame retardant chemicals to be used in furniture [18]. However, exposure is expected to continue for several decades because of the reservoir of these chemicals that exist in consumer products that have long durations of use (e.g. couches), and the environmental stability of PBDEs. The most recent data available from NHANES shows that a reduction in PBDEs in serum was not yet observed on a national level following the phase-out of several PBDEs in 2004 [19], although more current biomonitoring data suggest that levels are decreasing [20, 21].

We have known for over 20 years that some PBDEs are carcinogenic. A study by the National Toxicology Program reported in 1986 found that DecaBDE causes thyroid and liver tumors in rats and mice [22]. More recently, a NTP study on PBDEs found that DE71, the major commercial mixture of PentaBDE, is a two-species, two-sex carcinogen [23]. Epidemiological evidence demonstrates that PBDEs are also endocrine-disrupting compounds that interfere with thyroid hormone action [24], reproduction, and neurodevelopment [25–30]. Subtle changes in circulating thyroid hormone concentrations (e.g., free and total T_3_ (triiodothyronine), T_4_, TSH (thyroid stimulating hormone)) are associated with many adverse health effects. Thyroid hormones regulate the growth and differentiation of the brain and are therefore critical for neurodevelopment [31]; the developing fetus relies on maternal thyroid hormone until week 11 of gestation [32] before beginning to produce endogenous hormones, and doesn’t produce sufficient hormone for its own needs until approximately week 20 [33]. Further, because the thyroid is a modulator for many of the body’s organs, studies have linked thyroid conditions with other diseases like kidney [34], liver [35], and heart [36].

Thyroid disorders disproportionately impact women [37], and the rates of thyroid cancer are increasing globally, with women having higher rates of thyroid cancer than men, and older women having higher rates than younger women [38]. We know of only one study that evaluated PBDE-associated health effects in a representative U.S. population sample (NHANES) and those researchers found associations with diabetes and metabolic syndrome [39], but did not evaluate thyroid disease as an outcome. Further, we know of no research related to the potential sensitivity of menopausal women to thyroid disease based on PBDE concentration, despite the well-described role of estrogen on thyroid function [40] and the ability of PBDEs to exert estrogenic effects [41]. The objective of our research was to explore the relationship between PBDE exposures in women and thyroid disease using nationally-representative (U.S.) data from the NHANES.

## Methods

### Exposure Variable—PBDEs

At the time of publication, NHANES reported PBDE concentrations in serum for individuals (i.e., not pooled) for only one NHANES cycle: 2003–2004. We analyzed the data for the four PBDE congeners reported by NHANES (BDEs 47, 99, 100 and 153), all primarily associated with the PentaBDE commercial product; information on BDE 209, the primary congener of the DecaBDE commercial product, has yet to be reported in any NHANES cycle. Other researchers thoroughly describe the NHANES analytical methods for serum PBDE measurements and summary statistics for the population-weighted concentrations [42]. We created exposure categories based on quartiles of lipid-adjusted, sex-specific serum concentrations for each congener as reported by Sjodin et al. [42]. In addition, we generated a variable for total sum of lipid-adjusted PBDE concentrations, which combined the concentrations from the four congeners (ΣBDE).

### Outcome variable—thyroid disease

Thyroid hormone measurements are available in NHANES but did not overlap completely with the participants for whom PBDE serum measurements were also available. The NHANES questionnaire administered to all participants in NHANES 2003–2004 included two questions related to thyroid disease: MCQ160m “Has a doctor or other health professional ever told (you/SP) that (you/s/he) . . . had a thyroid problem?” and, MCQ170m “(Do you/Does SP) still . . . have a thyroid problem?”. Responses included: yes, no, don’t know or missing. For our analyses, if a subject responded “don’t know” then the data were considered missing. We used a combination of the thyroid questions to develop an outcome variable ‘current thyroid problem’. We defined having a current thyroid problem as an affirmative answer to ever having a thyroid problem and still have a thyroid problem. The sample size for participants with both PBDE measurements and thyroid disease status available are as follows: BDE 47, *n* = 1396; BDE 99, *n* = 1378; BDE 100, *n* = 1413; BDE 153, *n* = 1413.

### Thyroid disease prevalence

To calculate the survey-weighted prevalence of thyroid problems among the NHANES population, we generated weighted proportions by gender and post-menopause for questions MCQ160m (ever) and MCQ170m (current) according to the NHANES descriptive statistical methodology [43].

### Statistical analysis

We performed statistical analyses using STATA statistical software package, version 13.0 (StatCorp, College Station, TX). To estimate odds ratios and 95 % confidence intervals for development of disease accounting for the clustered sampling design in NHANES Control and Prevention (CDC), we used multivariate logistic regression modeling following procedures recommended by the Centers for Disease Control and Prevention [44]. We performed logistic regression analyses in this study using the STATA ‘svy’ commands to account for NHANES strata and cluster variables, and represent population-weighted results. To stratify the analysis to women-only, we assigned men a near zero weight in accordance with NHANES guidance. To stratify for women who were postmenopausal, we created a variable based on women who had not had one menstrual period in the last 12 months (RHQ031) and also indicated the reason for not having a period was post-menopause/hysterectomy (RHD042). Similar to the gender stratification, we assigned near zero weights to all men and women who were not postmenopausal.

We adjusted multivariate models for several potential confounders, using a similar methodology from a recent study examining thyroid effects in the NHANES study [45]. Covariates included in the model, with groupings in parentheses, were: race/ethnicity (Mexican American, other Hispanic, non-Hispanic White, non-Hispanic Black, other race (including multi-racial)); age (continuous variable); body mass index (BMI) (less than 25, 25–30, >30), education (less than high school, high school, more than high school), smoking (ever/never smoked more than 100 cigarettes in lifetime); alcohol consumption (never consume or at least one drink per week); and current hormone use (taking birth control pills now, now using Depo-Provera or injectables, taking estrogen only pills now, taking progestin only pills now, taking estrogen/progestin now, using estrogen-only patches now, using estrogen/progestin patch now).

In the primary models, the first and second quartile exposure groups (Q1 and Q2) were combined to increase statistical power and match the approach necessitated for BDE 99 (for BDE 99, the median serum concentration reported by Sjodin et al. [44] was ‘non-detect’), similar to the approach used by Melzer et al. [45].

## Results

In the 2003–4 NHANES survey, the prevalence of women reporting ever being told by a doctor that they had a thyroid problem was five times higher than the prevalence reported by men (16 % v. 3 %; p < 0.05). This was similar to the finding for those reporting a current thyroid problem (13 % v. 2 %; *p* < 0.05) (Table 1). In comparisons of postmenopausal and premenopausal women, we found that the prevalence of ever having a thyroid problem and the prevalence of currently having a thyroid problem was twice as high for postmenopausal women (ever: 24 % v. 12 %, *p* < 0.05; current: 18 % v. 10 %, *p* < 0.10).

**Table 1:** Survey-weighted prevalence of ever having a thyroid problem and having a current thyroid problem, by males v. females and premenopausal v. postmenopausal

Gender/Status	Prevalence (proportion)	Linearized SE	Lower 95 % CI	Upper 95 % CI
*Ever had a thyroid problem*				
All men (*n* = 2414)	0.03	0.009	0.02	0.06
All women (*n* = 2613)	0.16	0.017	0.13	0.20
Premenopausal women (*n* = 1070)	0.12	0.025	0.07	0.18
Postmenopausal women (*n* = 1144)	0.24	0.026	0.19	0.30
*Current thyroid problem*				
All men (*n* = 2410)	0.02	0.009	0.01	0.05
All women (*n* = 2594)	0.13	0.015	0.10	0.17
Premenopausal women (*n* = 1065)	0.10	0.022	0.06	0.16
Postmenopausal women (*n* = 1.131)	0.18	0.027	0.13	0.24

In fully-adjusted, survey-weighted logistic regression models we found that women in the highest quartile of serum concentrations for BDE 47, 99, 100 and ΣBDE, had a greater odds of currently having a thyroid problem, compared to women in the lower quartiles of serum concentrations (Table 2); statistically significant associations were observed for BDE 47 (OR: 1.48; 95 % CI: 1.05–2.09), BDE 99 (OR: 1.78; 95 % CI: 1.16–2.75) and ΣBDE (OR: 1.61; 95 % CI: 1.1–2.4), and marginally significant for BDE 100 (OR: 1.50; 95 % CI: 0.97–2.31). The point estimates of the odds ratios for the 3rd quartile were less than unity, although only statistically significant for the ΣBDE variable.

**Table 2:** Fully-adjusted, survey-weighted associations between serum PBDE concentrations and having a current thyroid problem. Adjusted for: age, race/ethnicity, BMI, education, smoking, alcohol consumption, and current hormone use

	BDE47 *n* = 1075		BDE99 *n* = 1061		BDE100 *n* = 1089		BDE153 *n* = 1089		Sum BDEs *n* = 1061	
	OR	(95 % CI)	OR	(95 % CI)	OR	(95 % CI)	OR	(95 % CI)	OR	(95 % CI)
Men and Women Combined										
Q1 + Q2 (ref)	1		1		1		1		1	
Q3	0.55	(0.20–1.49)	0.93	(0.38–2.27)	0.74	(0.44–1.25)	0.92	(0.30–2.84)	0.57	(0.23–1.39)
Q4	1.32	(0.90–1.96)	1.22	(0.80–1.84)	1.13	(0.79–1.60)	1.14	(0.56–2.31)	1.09	(0.71–1.67)
	BDE47 n = 512		BDE99 n = 504		BDE100 n = 518		BDE153 n = 518		Sum BDEs n = 504	
All Women										
Q1 + Q2 (ref)	1		1		1		1		1	
Q3	0.60	(0.19–1.85)	0.75	(0.27–2.08)	0.65	(0.27–1.54)	1.00	(0.27–3.78)	0.36**	(0.15–0.89)
Q4	1.48**	(1.05–2.09)	1.78**	(1.16–2.75)	1.50*	(0.97–2.31)	1.44	(0.68–3.05)	1.61**	(1.10–2.36)
	BDE47 n = 236		BDE99 n = 233		BDE100 n = 240		BDE153 n = 240		Sum BDEs n = 233	
Postmenopausal Women										
Q1 + Q2 (ref)	1		1		1		1		1	
Q3	0.81	(0.24–2.71)	0.99	(0.34–2.93)	0.23**	(0.07–0.68)	0.50	(0.13–1.90)	0.54	(0.15–1.97)
Q4	2.15*	(0.86–5.37)	3.63**	(1.12–11.8)	2.02*	(0.93–4.36)	1.25	(0.49–3.21)	2.20*	(0.92–5.26)

***p < 0.01,**p < 0.05, *p < 0.1

For all PBDE congeners except for BDE 153, the odds ratios for women in the highest exposure category were higher when the analysis was restricted to postmenopausal women. For example, for BDE 47, when all women were included in the analysis, the odds of having a current thyroid problem was 1.5 compared to 2.1 for just postmenopausal women. For BDE 99, this difference was two-fold, with postmenopausal women in the highest exposure category having 3.6 times the odds of having a current thyroid problem (95 % CI: 1.1–11.8), compared to 1.5 times the odds observed for all women (95 % CI: 0.97–2.3). The analysis for a current thyroid problem post-menopause showed odds ratios that were less than unity for the 3rd quartile.

The NHANES dataset did not contain enough men with current thyroid disease for our models to converge in a men-only analysis. In an analysis with all men and women combined, we did not find significant associations between the exposure categories and current thyroid disease (Table 1). Similarly, we originally attempted to explore thyroid cancer specifically, but this NHANES cycle only had nine participants reporting thyroid cancers, precluding us from performing regression analysis.

## Discussion

Our study found a positive association between PBDEs in serum and thyroid disease in U.S. women, a finding that suggests that effects of PBDEs on thyroid hormones, documented extensively in toxicological and epidemiological studies, is leading to significant downstream impacts. For example, increased PBDE levels in animals, primarily in mice and rats studies, have been associated with altered T_4_ and T_3_ levels [46–50] suggesting thyroid dysregulation *in vivo*, with several proposed mechanisms of action [51–54]. Associations between thyroid hormone levels and PBDEs have also been observed for animals in the wild, including birds, fish, and polar bears [55–57]. In humans, there are many studies that describe similar findings of associations between PBDE concentrations in serum and thyroid hormone concentrations [21, 25, 26, 29, 58–61].

One possible mechanism accounting for the PBDE-induced reduction in serum T_4_ is displacement of T_4_ from the serum binding protein transthyretin (TTR) or thyroxine binding globulin (TBG) [54]—similar to what has been previously observed for polychlorinated biphenyls (PCBs) [62]. (Significantly, a study examining the effects of chemical mixtures observed a potentially synergistic effect on T_4_ levels with co-exposures to BDE 47 and PCBs [47]). T_3_ and T_4_ travel in blood bound to plasma proteins; only 0.04 % of T_4_ and 0.4 % of T_3_ are unbound (or “free”), and consequently, available for entry and action in target tissues [32]. The tetra-brominated congeners, BDE 47 in particular, share a similar structure to T_4_, with both having the diphenyl ether structure and four halogens (iodine for T_4_; bromine for BDE47). A major difference is that T_4_ has a hydroxyl group on one of the rings, positioned between the two halogens. However, PBDEs are hydroxylated in vivo and, when this occurs in the meta position, it yields 3-OH-BDE47, which also has the hydroxyl group positioned between two halogens [53].

Along with epidemiological and toxicological evidence showing perturbations in thyroid hormone concentrations, we are now beginning to understand plausible mechanisms by which this disruption is occurring; the similarity of structures of exogenous chemicals to endogenous hormones may be leading to competition for receptor binding sites, causing inhibition or amplification. For thyroid hormones, a study by Cao et al. [52] found that hydroxylated PBDEs bind to transthyretin (TTR) and thyroxine-binding globulin (TBG) at the same sites as the target hormone, T_4_. Hamers et al. measured binding affinities for PBDEs and metabolites to TTR and reported that the hydroxylated metabolites bound 160–1600 times more avidly than the parent compounds [63]. Further, a study by Butt and Stapleton [51] found that hydroxylated PBDEs are potent inhibitors of thyroid hormone sulfation. Also relevant to our study that focused on menopausal status, hydroxylated PBDEs have been shown to compete with estrogen enzymes. A crystallographic analysis examining the binding affinity of 3-OH-BDE47 to a hormone enzyme, estrogen sulfotransferase, demonstrated that the first phenolic group positions the compound within the enzyme similarly to how 17βestradiol would situate [41]. Further, the authors demonstrated that this positioning creates additional hydrogen bonding via the hydroxyl group, also similar to 17βestradiol, while accommodating the two halogens.

Perhaps the most striking and unique finding in this study is that the odds of having a current thyroid problem associated with PBDEs are so much higher in postmenopausal women. One hypothesis is that this is related to the change in hormone concentrations in postmenopausal women and the affinity of PBDEs to binding sites for both estrogen and thyroid hormones. Menopause is initiated when the ovaries discontinue producing two hormones, estrogen and progesterone. In at least two ways, the strong binding affinity of OH-PBDEs with estrogen sulfotransferase can interfere with the change in estrogen levels that occur during menopause. First, with less estrogen in the system, there may be increased metabolism of PBDEs by this estrogen enzyme [41]. Second, the OH-PBDEs may be competing for these binding sites that are critical for the removal of circulating estrogen produced by other tissues, leading to higher than expected levels of circulating estrogen. This potential interference of the estrogen clearance pathway in the liver via estrogen sulfotransferase, in and of itself, would not explain the higher odds of thyroid problems. However, estrogen (and androgen) can increase the levels of serum thyroxine binding globulin (TBG), interfering with one of the three primary proteins responsible for thyroid hormone transport in serum. In addition to these potential impacts on thyroid hormone via estrogen-mediated pathways, PBDEs can also have a direct impact on thyroid hormones through serum binding proteins outside of the mechanism that involves estrogen; PBDEs show a strong affinity for both TBG and another serum binding protein, transthyretin, and therefore can have a more direct impact on circulating thyroid hormone levels [41, 52]. All of this suggests the disruption of thyroid signaling by PBDEs that may be enhanced by the altered estrogen levels during menopause.

Results from epidemiologic studies on the effects of PBDEs on thyroid hormones are consistent with the toxicological evidence, in that they suggest interference with thyroid hormone regulation. However, in some epidemiologic studies, estimates of PBDE exposure are positively associated with measures of thyroid function, and in others the opposite pattern is observed. For example, in a study of 297 infants, Herbstman et al. observed that cord blood serum PBDE levels were associated with reduced total and free T_4_ levels [27]. Conversely, Turyk et al. (2008) conducted a study of 405 adult male sport fish consumer and observed a positive trend between free T_4_ and PBDE serum levels [61]. A more recent study found a positive association between PBDEs in serum of pregnant women and serum total and free T_4_ and T_3_ [29].

There are seeming inconsistencies, then, in the epidemiological literature, with some studies showing positive associations with PBDEs and thyroid hormones and others showing negative associations. We note that the PBDE serum concentrations in many of these cohort studies are in different ranges of the concentration distribution found in the U.S. population, with some representing the low-end of exposure and others on the high-end (selected studies; Table 3) [21, 25, 26, 29, 58–60]. Moreover, it seems reasonable to consider the possibility that adults and fetuses have many differences in thyroid physiology that could account for at least some of these differences. However, we hypothesize that a potential explanation of the differences in findings is due to: 1) non-monotonic dose responses to PBDEs, and 2) where on the dose–response range the studies were performed. Within our study using NHANES data, which represents the full range of population exposures in the U.S. and therefore in theory encompasses the ranges found in these other studies, we also observed that the shape of the response curve is non-linear and suggests a non-monotonic dose response curve (NMDRC) may exist (e.g. Q1 + Q2 have higher odds of current thyroid problem relative to Q3; Q4 also has higher odds of current thyroid problem relative to Q3). Vandenberg et al. [64] published a seminal paper evaluating the literature related to endocrine disrupting chemicals and low-dose and nonmonotonic dose responses, with significant implications to our current approach to protecting public health [65]. Vandenberg et al. state that NMDRCs are, “not the exception, but should be expected and perhaps even common,” and that it is no longer acceptable to dismiss NMDRCs based on a lack of mechanism because there are now several potential mechanisms to explain these phenomena [64]. These include cytotoxicity (toxic at high concentrations and biologically active at low concentrations), cell- and tissue-specific receptors and co-factors, receptor sensitivity, and receptor down-regulation and desensitization, among other proposed mechanisms. In our analysis, individuals in the first and last quartiles (Q1 + Q2 and Q4) have higher odds of a thyroid problem, compared to Q3. Results from the other study of PBDEs using NHANES that focused on diabetes and metabolic syndrome were also suggestive of a NMDRC for BDE 99 [39]. It is possible that the existence of a NMDRC for PBDEs may explain the seeming inconsistency across studies examining the relationship between PBDEs and thyroid hormones. The serum concentrations in these studies generally fall within very different ranges of the full distribution of serum concentrations observed in NHANES (Table 3). For example, the central tendency PBDE concentrations in Zota et al. [21] fall within the Q4 of NHANES, and they found that PBDEs were associated lower free T_4_. In contrast, the central tendency PBDE concentrations in Abdelouahab et al. [25] fall within Q3 of NHANES, and they found that PBDEs were associated with the opposite effect (higher free T_4_). More research is needed to fully explore this hypothesis.

**Table 3:** Selected studies reporting associations between serum BDE 47 concentrations (ng/g lipid) and associations with thyroid hormone, in relation to NHANES serum concentrations

NHANES (all participants)	25th percentile 9.3			50th percentile 19.2				75th percentile 40.9
Epi Study	Hagmar 2001 [60]	Dallaire 2009 [59]	Bloom 2008 [58]	Chevrier 2010 [26]	Turyk 2008 [61]	Stapleton 2011 [29]	Abdelouahab 2013 [25]	Zota 2011 [30]
n	110	623	36	270	405	140	380	25
Gender	Men	Men	Men (mostly)	Women (pregnant)	Men	Women (pregnant)	Women (pregnant)	Women (pregnant)
Central Tendency BDE 47	1.04	2.16	7.9	15.3	16.2^#^	16.5	21.5	43.1
TSH	-*	-	-	-*	-	+	+	+
Total T_3_		+*			-*	+	-*	
Free T_3_					-	+	+	
Total T_4_				-	-	+*	-*	+
Free T_4_		+	+	+	+	+*	+*	-

+ positive association

- Negative association

* *p* < 0.05

# estimated

A limitation of our study is the inability of this analysis to determine causality because the data are cross-sectional; thus, we do not have temporality captured in the data to know if the exposure preceded the outcome. Also, the standard approach to analysis of serum concentrations for lipophilic compounds is to adjust the concentrations based on lipid-content in the serum and report the results as ‘lipid-adjusted’. This approach, however, may have a critical limitation. Thyroid hormones affect lipid metabolism at several points—synthesis, mobilization and degradation [66]. If a person has thyroid disease not caused by PBDE exposure, and the disease increases lipid metabolism leading to lower lipids in the bloodstream, then adjusting PBDEs by this lower lipid concentration yields a higher lipid-adjusted PBDE concentration, creating the appearance of a positive association between thyroid disease and PBDEs. Therefore, it is possible that women who self-report current thyroid disease have elevated lipid-adjusted PBDE concentrations in serum as a result of having thyroid disease. Further, even if serum concentrations are not adjusted for lipid content, the concentration in serum is still impacted by lipid content in the bloodstream, so a simple solution cannot be using serum concentrations that are not lipid-adjusted (we performed an analysis using wet-weight concentrations and found similar results as when we used lipid-normalized concentrations; Additional file 1: Table S1). Considering these points, it is plausible then that the relationship of thyroid dysfunction to PBDEs could be a function of reverse causality, with PBDE concentrations, lipid-adjusted or not, simply an outcome of thyroid dysfunction and altered lipid metabolism.

Another potential limitation of this study is the lack of specificity in the NHANES questionnaire regarding thyroid problems. NHANES is a nationally representative health survey (U.S.) that, due to its breadth, only covers thyroid-related disease with three non-specific questions: 1) Has your doctor ever told you that you have a thyroid problem, 2) Do you still have a thyroid problem, and 3) Do you have thyroid cancer. Yet, ‘thyroid problem’ and ‘thyroid cancer’ encompass a wide range of specific illnesses (e.g., hyperthyroidism, hypothyroidism, nodules, thyroiditis, goiter; papillary thyroid cancer, follicular thyroid cancer, medullary thyroid cancer), each with their own potential etiology (e.g., iodine deficiency, Graves’ disease, Hashimoto’s disease, radiation, environmental chemicals). Despite the lack of specificity in the NHANES questions on the outcome variables, which would likely bias the results toward a null finding, we still observed strong and consistent associations between PBDEs in serum and thyroid problems. Last, the congeners available for analysis in NHANES are also from the same commercial product and are correlated in environmental and body burden samples [5, 42]. Therefore, we cannot rule out that one congener is driving the observed associations seen for several congeners.

Overall, the findings from this analysis of data reported in NHANES provide additional evidence of the impact of PBDEs on the thyroid. We also observed a stronger effect for postmenopausal women. Additional research is warranted to confirm these findings, particularly when the next cycle of NHANES data with PBDE serum concentrations is made available.

## Conclusion

We found that women with the highest flame retardant concentrations in their blood were significantly more likely to have a thyroid problem, with stronger associations found for post-menopausal women. Specifically, women with lipid-adjusted serum concentrations of BDE 47, 99 and 100 in the highest quartile of exposure had higher odds of having a current thyroid problem compared to women with lower serum concentrations. These associations were much stronger in an analysis that was restricted to postmenopausal women.

### Ethics

This research did not require ethics approval by an Institutional Review Board (IRB) as the data are de-identified data from the National Health and Nutrition Examination Survey (NHANES), a publicly available dataset.

## Additional file

Additional file 1: Table S1. Description of Data: Fully-adjusted, survey-weighted associations between serum PBDE concentrations (wet weight) and having a current thyroid problem. (PDF 669 kb)

## Abbreviations

ΣBDE: Sum of BDEs 47, 99, 100,183
BDE 47: 2,2′4,4′-tetra-bromodiphenyl ether
BDE 99: 2,2′4,4′5-penta-bromodiphenyl ether
BDE 100: 2,2′,4,4′,6-penta-bromodiphenyl ether
BDE 183: 2,2′,3,4,4′,5′,6-hepta-bromodiphenly ether
BDE 209: deca-bromodiphenyl ether
BMI: body mass index
CDC: Centers for Disease Control and Prevention
FR: flame retardant
NHANES: National Health and Nutrition Examination Survey
NMDRC: non-monotonic dose response curve
NTP: National Toxicology Program
OR: odds ratio
PBB: polybrominated biphenyl
PBDE: polybrominated diphenyl ether
PCB: polychlorinated biphenyl
T_3_: triiodothyronine
T_4_: Thyroxine
TBBPA: Tetrabromobisphenol A
TBG: thyroxine-binding globulin
TSH: thyroid stimulating hormone
TTR: transthyretin
