# Supplementary material for: PBDE flame retardants, thyroid disease, and menopausal status in U.S. women
Source: Environ Health. 2016 May 24;15:60. doi: 10.1186/s12940-016-0141-0 (PMC4877989; doi:10.1186/s12940-016-0141-0)
Supplement: Additional file 1: Table S1. — Description of Data: Fully-adjusted, survey-weighted associations between serum PBDE concentrations (wet weight) and having a current thyroid problem. (PDF 669 kb) [file 12940_2016_141_MOESM1_ESM.pdf]

Additional File 1

Table S1. Fully-adjusted, survey-weighted associations between serum PBDE concentrations (wet weight) and having a current thyroid problem

	**BDE47**		**BDE99**		**BDE100**		**BDE153**		**Sum BDEs**	
	OR	(95% CI)	OR	(95% CI)	OR	(95% CI)	OR	(95% CI)	OR	(95% CI)
**Men and Women Combined**										
**Q1 + Q2 (ref)**	1		1		1		1		1	
**Q3**	0.56	(0.26 - 1.17)	0.82	(0.49 - 1.37)	0.55	(0.26 - 1.17)	0.85	(0.39 - 1.87)	0.60	(0.29 - 1.24)
**Q4**	1.24	(0.77 - 2.00)	1.18	(0.78 - 1.79)	0.96	(0.63 - 1.47)	0.91	(0.53 - 1.56)	1.2	(0.75 - 1.85)
**All Women**										
**Q1 + Q2 (ref)**	1		1		1		1		1	
**Q3**	0.42	(0.08 - 2.16)	0.70	(0.33 - 1.52)	0.48	(0.17 - 1.35)	1.01	(0.30 - 3.41)	0.33*	(0.10 - 1.04)
**Q4**	1.41	(0.89 - 2.22)	1.80**	(1.16 - 2.81)	1.25	(0.86 - 1.81)	1.38	(0.69 - 2.77)	1.55**	(1.08 - 2.22)
**Postmenopausal Women**										
**Q1 + Q2 (ref)**	1		1		1		1		1	
**Q3**	0.71	(0.13 - 3.78)	0.77	(0.30 – 2.00)	0.28**	(0.10 - 0.84)	0.47	(0.17 - 1.27)	0.24**	(0.06 - 0.91)
**Q4**	2.17	(0.73 - 6.40)	3.50**	(1.12 - 10.9)	1.88*	(0.91 - 3.91)	1.01	(0.40 - 2.55)	2.13	(0.80 - 5.69)

***p<0.01,**p<0.05, *p<0.1

Adjusted for:

Age, race/ethnicity,BMI, education, smoking, alcohol consumption, and current hormone use (only in the female analyses).
